# High-Hydrostatic-Pressure (HHP) Processing Technology as a Novel Control Method for *Listeria monocytogenes* Occurrence in Mediterranean-Style Dry-Fermented Sausages

**DOI:** 10.3390/foods8120672

**Published:** 2019-12-12

**Authors:** Domenico Meloni

**Affiliations:** Department of Veterinary Medicine, University of Sassari, Via Vienna 2, 07100 Sassari, Italy; dmeloni@uniss.it; Tel.: +39-079-229-570; Fax: +39-079-229-458

**Keywords:** high hydrostatic pressures, *Listeria monocytogenes*, Mediterranean, fermented sausages, hurdles technology

## Abstract

Although conventional microbial control techniques are currently employed and largely successful, their major drawbacks are related to their effects on quality of processed food. In recent years, there has been a growing demand for high-quality foods that are microbially safe and retain most of their natural freshness. Therefore, several modern and innovative methods of microbial control in food processing have been developed. High-hydrostatic-pressure (HHP) processing technology has been mainly used to enhance the food safety of ready-to-eat (RTE) products as a new pre-/post-packaging, non-thermal purification method in the meat industry. *Listeria monocytogenes* is a pertinent target for microbiological safety and shelf-life; due to its capacity to multiply in a broad range of food environments, is extremely complicated to prevent in fermented-sausage-producing plants. The frequent detection of *L. monocytogenes* in final products emphasizes the necessity for the producers of fermented sausages to correctly overcome the hurdles of the technological process and to prevent the presence of *L. monocytogenes* by applying novel control techniques. This review discusses a collection of recent studies describing pressure-induced elimination of *L. monocytogenes* in fermented sausages produced in the Mediterranean area.

## 1. Introduction

In recent years, food business operators (FBOs) have been repeatedly required to meet the request of consumers for more unadulterated, preservative-free, and less-processed food products, without losing their microbiological quality and safety [1]. Although thermal treatments are a powerful technology for the elimination of foodborne pathogens in foods, they can cause alterations of texture and color [2]. Non-thermal technologies, for instance high hydrostatic pressures (HHP), have emerged as new preservation techniques to inactivate the foodborne pathogens present in foods and consequently improve their shelf life [3]. 

HHP essentially involves the application of isostatic pressures, equally and immediately transmitted to food products by air-driven pumps through a liquid [4]. HHP processing technologies have demonstrated a high capacity to eliminate foodborne pathogens and spoilage bacteria, leading to foods with better microbiological quality and safety [5]. 

HHP has primarily been used in the ready-to-eat (RTE) meat products industry as a new pre-/post-packaging, non-thermal decontamination method [6]. The implementation of HHP has been proposed to enhance the safety of dry-fermented sausages, since thermal methods may have unsatisfactory effects on their quality [7,8]. In these products, the application of HHP could relieve the use of nitrites without negative effects on food safety [9,10]. Nitrites are routinely used to avoid toxin production by *Clostridium botulinum*. Furthermore, in fermented meat products, nitrites and/or nitrates are well known for their antagonistic outcomes against the main foodborne pathogens. [11,12,13,14]. In the European Union, the quantity of nitrates and nitrites allowed in preserved meat products is outlined in the EC Reg.1129/2011: the permitted quantity is 250 ppm NaNO_3_ for traditional products without addition of nitrites, or 150/150 ppm NaNO_3_/NaNO_2_ in different cases [15]. Even though the advantages of preservatives are noted, the use of nitrites in meat preservation has caused public concern, since nitrites can be precursors of nitrosamines, many of which are well known to be carcinogenic agents [16]. Therefore, consumers and FBOs are pushing for the development of products with lower amounts of nitrates/nitrites, and some countries are debating a reduction of allowed quantities [15]. 

Numerous studies have investigated the use of HHP to avoid the presence of *L. monocytogenes* in several meat products [5,17,18,19,20,21,22,23,24]. HHP has been shown to alter the structure, morphology, and physiology of *L. monocytogenes*. The effects are related to food composition, time of submission, amount of pressure, and existence of antimicrobial substances [25,26].

*L. monocytogenes* is a small, non-spore-producing, permissive, anaerobic, Gram-positive rod. Peritrichous flagella enable a characteristic whirling mobility at 20–25 °C. In relation to the presence of somatic (O) and flagellar (H) antigens, thirteen serogroups have been determined: 1/2a, 1/2b, 1/2c, 3a, 3b, 3c, 4a, 4ab, 4b, 4c, 4d, 4e, and 7. *L. monocytogenes* is catalase-positive, oxidase-negative, and grows between 0 and 45 °C, with an optimum temperature at 30–37 °C.

The pathogen survives at pH values between 4.5 and 9.0 (optimum pH 6–8), and can grow in foods with a water activity (a_w_) of 0.92. *L. monocytogenes* is ubiquitous in the ecosystem; the main contamination sources are soil, forage, and water [27,28]. Other reservoirs include healthy humans [29] or contaminated farm and wild animals [30]. *L. monocytogenes* can multiply at low temperatures, survives under modified atmospheres, and adheres to several surfaces, adapting to disinfectants [31]. Moreover, *L. monocytogenes* can resist NaCl (up to 12%) and nitrates, which are commonly lethal to other bacteria [32,33,34]. 

*L. monocytogenes* is widespread in food-processing plants; once introduced, it can grow in biofilms and severe conditions, becoming a primary niche of contamination [35,36,37,38]. *L. monocytogenes* is the etiological cause of human listeriosis. This serious invasive disease is nearly completely caused by *L. monocytogenes*. Uncommon episodes are caused by *L. ivanovii* and *L. seeligeri* [32]. *L. monocytogenes* isolates differ in their pathogenic potential; several strains are innately virulent, causing elevated morbidity and mortality cases, while other strains are non-virulent and not capable of causing illness in humans and animals [32]. Differentiation between virulent and non-virulent isolates is fundamental to evaluating the impact of *L. monocytogenes* on public health [32]. 

*L. monocytogenes* strains have been grouped into three evolutionary lineages differentiated by diverse pathogenic potential: Lineage I includes isolates related to listeriosis outbreaks (serogroups 1/2b, 3b, 4b, 4d, and 4e); Lineage II consists of isolates associated with sporadic human cases (serogroups 1/2a, 1/2c, 3a, and 3c); and Lineage III includes isolates hardly ever connected with listeriosis outbreaks or sporadic episodes. Most of the listeriosis outbreaks or sporadic episodes are known to be food-borne, and have an impact on the central nervous and circulatory systems and the gravid female uterus. Two types of human listeriosis have been reported, with symptomatology which includes febrile gastroenteritis in healthy people [39], and septicemia and meningoencephalitis in risk categories, such as young, old, pregnant and immune-compromised people, the so-called “YOPI” [40,41]. 

Listeriosis is the fifth most common zoonotic disease in the European Union, with an annual incidence of 0.48 cases per 100,000 population and 2480 confirmed cases mostly acquired domestically [30]. The highest rates have been reported in Finland, Denmark, Germany, Luxembourg, Sweden, and Belgium, with 1.62, 1.01, 0.88, 0.85, 0.81, and 0.80 cases per 100,000 population, respectively [30]. The lowest rates (≤0.2 per 100,000) have been reported by Bulgaria, Croatia, Cyprus, Malta, and Romania [30]. 

The highest infection degrees have been outlined in elderly people, mainly aged above 84 years [42]. Listeriosis has the highest hospitalization degree of all zoonoses under European Union surveillance, and 98.6% of occurrences show additional long-term sequelae [30]. Listeriosis is also the first predominant cause of death in the European Union, with an approximated case fatality rate of 13.8% [30]. Altogether, 277 deaths were recorded in 2017, the highest number of lethal episodes described since 2006 [30]. 

The morphology, physiology, and epidemiology of *L. monocytogenes*, combined with the harshness of human listeriosis, give the presence and the enumeration of the pathogen a certain importance for FBOs of cold-stored RTE food products. *L. monocytogenes* has been isolated from several RTE foods [43,44,45] and has been associated with a wide number of outbreaks involving RTE meat, poultry, dairy, fish, and vegetable products [28,46,47]. 

In the European Union, the food safety criteria for *L. monocytogenes* in RTE food products are published in the EC Reg. 2073/2005. In RTE foods designated for infants and for special medical purposes, the absence of *L. monocytogenes* in 25 g is mandatory. In contrast, in RTE foods able to support the growth of *L. monocytogenes* (pH ≤ 4.4 or a_w_ ≤ 0.92 or pH ≤ 5.0 and a_w_ ≤ 0.94), *L. monocytogenes* cannot exceed 100 CFU/g throughout the product’s shelf life. In the same food products, the absence of *L. monocytogenes* in 25 g at the production stage is mandatory. However, the FBOs must confirm that these foods will not exceed the 100 CFU/g during their shelf life. In 2017, the highest level of non-compliance in several RTE foods was reported at the processing stage [30]. The uppermost levels of non-compliance were reported in fish and fishery products (0.2–3.9%), followed by soft and semi-soft cheeses (0.1–2.5%) and other dairy products (0–1.5%). At retail, *L. monocytogenes* occurrence was uppermost in fish and fishery products (6%), followed by salads (4.2%), meat and meat products (1.8%), soft and semi-soft cheeses (0.9%), fruit and vegetables (0.6%), and hard cheeses (0.1%) [30]. Although a small number of batches of fermented pork sausages were examined and none were positive, fermented sausages contaminated by *L. monocytogenes* at >100 CFU/g and consumed without further heat treatment should be considered a great public health concern [30]. 

In recent years, much efforts have been put into developing HHP processing technology in order to ascertain the most effective inactivation kinetics of *L. monocytogenes* in HHP-processed foods [20,48,49,50]. This review presents a discussion of recently published data focusing on pressure-induced elimination of *L. monocytogenes* in meat products, mainly fermented sausages produced in the Mediterranean area. 

## 2. *Listeria monocytogenes* in the Pork Meat Processing Industry

### 2.1. Swine Slaughterhouses

The presence of *L. monocytogenes* has been reported in all the stages of the meat supply chain [51,52,53], including swine slaughterhouses [54,55,56]. Even though swine are well known not to be the main carriers of *L. monocytogenes*, pork meat is extremely vulnerable to contamination by *L. monocytogenes* [57,58,59]. 

The main routes of *L. monocytogenes* contamination can be animal-related or through the slaughterhouse environment [60,61]. Due to the ubiquity of *L. monocytogenes* in swine slaughterhouses, swine carcasses can be contaminated at any stage [61]. *L. monocytogenes* is propagated to the carcasses from the pigs; it has been periodically found in feces and on the surface of animals in good health with the pathogen present in the intestines [62,63]. 

The occurrence in feces can reach 50%. Pigs with positive cecal content could have been infected on the farm or throughout transport or the pre-slaughtering interval in highly contaminated lairage areas, which can be considered the primary sources for *L. monocytogenes* contamination prior to slaughter [56,60,64,65,66]. 

The role of pigs as a niche of environmental contamination has been well demonstrated; the area of stunning/hanging is generally the most contaminated [67,68,69,70]. The highest prevalence of *L. monocytogenes* is usually found at the end of dressing, due to handling of the carcasses with contaminated utensils and equipment [63,71]. Several authors [65,66] have highlighted that the contamination of equipment is related to the occurrence of *L. monocytogenes* in the tongue (14%) and tonsils (7–61%). At the time of evisceration [72,73], the contamination of carcasses by *L. monocytogenes* can be around 60%–65% [53,60,74]. In slaughterhouse environments, surfaces in contact with meat such as knives, carcass splitters, or dehairing equipment are the most highly contaminated areas and are crucial sites for the cross-contamination of carcasses [56]. The prevalence of *L. monocytogenes* in meat non-contact surfaces such as floor drains is generally around 20%–30%, and is probably correlated with inadequate cleaning procedures [56]. Even though floor drain water is not contemplated as a critical control point, carcass contamination can occur if contaminated water is sprayed at high pressure during cleaning procedures with the consequent formation of aerosols [56]. 

The monitoring over time of the contamination of slaughterhouses over consecutive sampling sessions can be a useful tool to identify the emergence of new subtypes, as well as the persistence of resident “house strains” [75,76,77]. Some serogroups of *L. monocytogenes* can persist in the same ecological niches in the slaughterhouses for months or years, becoming endemic and plant-specific through the formation of biofilms [56]. The most common recurrent serogroups in swine slaughterhouses are 4b, 1/2a, and 1/2c [56,65,74,78], while serogroup 1/2b is generally less frequent.

### 2.2. Meat Processing Plants

Previous authors have reported that the degree of *L. monocytogenes* contamination increases all along the meat production chain [53,79]. The most important magnification niche is the cross-contamination in meat-processing plant environments [63,80,81,82]. Raw meat is the major source of contamination; a high prevalence is generally found (45%–50%), compared to the tissues of recently slaughtered animals (0%–2%) [66,74,80,83]. In general, reception areas, refrigeration, and processing rooms are the most contaminated environments [79]. During the chilling and deboning stages, the presence of *L. monocytogenes* in chopped meat ranges between 16% and 50% [79,84]. The amount of contamination increases importantly up to 70%–100% during grinding and bagging [51,74]. At the same time, the occurrence of *L. monocytogenes* on surfaces in contact and without contact with meat varies between 17% and 50% and 11% and 25%, respectively [74,85].

*L. monocytogenes* can survive in harsh physical and chemical conditions, forming biofilms on surfaces within the food-processing environment [32,53,86,87]. In meat-processing plants, all surfaces and materials are expected to be contaminated by *L. monocytogenes* (e.g., pipelines, knifes, hooks, gaskets, conveyor belts cutters, slicers, brining and packaging machines, coolers, and freezers, as well as floors and drains) if sanitation is inadequate and/or infrequent. In addition, the more the surfaces show probable harborage niches (hollow parts, crevices, cracks, unpolished or worn materials), the more challenging they are to clean and disinfect and the more *L. monocytogenes* can settle and colonize them. The capacity of *L. monocytogenes* to grow in biofilms is thus fundamental for its persistence in food-processing facilities and is well known that these biofilms are the major source of contamination [57,58,59]. 

Meat-processing plants represent a suitable environment for the growth of *L. monocytogenes* biofilms; organic residues can be a niche for its accumulation and biofilm formation, as they are a source of cross-contamination [88]. In particular, meat grinders, worktables, and floor drains can be crucial harborage niches in the meat-processing plant environment [89,90,91,92,93]. In addition, at low temperatures, *L. monocytogenes* can survive and even increase in numbers as time progresses [58]. *L monocytogenes* can adhere speedily and tightly to inert surfaces such as metal, glass, rubber, and plastic [57,58,59]. Previous studies [85,87,91] have shown a persistence of 3–4 months of *L. monocytogenes* strains with weak or moderate capacity to form biofilms on polystyrene surfaces. The most frequent serogroups of *L. monocytogenes* found in meat-processing environments are 1/2c and 1/2a, while serogroups 1/2b and 4b are generally less frequent. [52,79,85,91]. In general, serogroups 1/2a, 1/2b, and 4b have shown greater capacity to form biofilms than serogroup 1/2c [91]. 

Biofilms are a perpetual niche of contamination for foods, and it has been frequently shown that adhered *L. monocytogenes* strains are more resistant to stress than the planktonic cells [89,90]. *L. monocytogenes* entrapped in biofilms is afforded unusual protection against sanitizers and disinfectants [91,94,95]; previous surveys have shown that the suggested concentrations of sanitizers are greater than necessary [38]. Decreases of *L. monocytogenes* growth in vitro after 24, 48, and 72 h of exposition to chlorines and quaternary ammonium at 37% concentration have been previously reported [85]. 

## 3. Production of Fermented Sausages

The manufacturing of fermented sausages in the Mediterranean area is a dynamic process characterized by biochemical, microbiological, physical, and sensorial changes occurring in minced ground meat formulation during fermentation and ripening [96]. Mediterranean-style dry-fermented sausages are identified by a long shelf life and a generally low sanitary risk due to their low pH (< 4.5–5) and a_w_ (<0.90), salt, and nitrites, chemical preservatives. [40,97,98]. In “artisanal” sausages, the microbial fermentation is due to natural bacterial microflora consisting mainly of coagulase-negative staphylococci (CNS) and lactic acid bacteria (LAB). In “industrial” sausages, microbial starters are added (especially LAB), in association with powdered milk in order to boost the process of acidification and thus maturation [98]. 

The safety of fermented sausages is guaranteed by the combination of several environmental parameters, known as “hurdle technology” [99]. The “hurdle technology” concept includes a sequence of different essential hurdles at sequential stages of fermentation and ripening [100]. The synergistic and complementary combination of pH and a_w_ prevent colonization and/or prevent the growth of foodborne pathogens [101]. The simultaneous application of two or more hurdles at low levels provides the same effect that would be achieved by the application at higher levels of a single hurdle [100]. As a result of their sequential application, pathogens and spoilage microorganisms are successfully prevented in fermented sausages, leading to selection for the expected antagonistic microflora, mainly LAB [101]. These hurdles are essential during the fermentation and ripening stages and lead to stable and safe ripened products [99]. 

The microbial ecosystem of meat fermentation includes a complex and multispecies population; in the early steps, nitrite-curing salts inhibit *Pseudomonas* bacteria and other Gram-negative microorganisms, which easily grow and damage non-preserved meat in aerobic conditions [99]. Therefore, LAB and CNS are the prevalent microflora coexisting with enterococci, yeasts, and molds [102,103]. In general, CNS are prevalent in the early stages of processing. 

At the end of maturation, CNS are the second microbial group after LAB [97]. CNS play a fundamental role in the maturation stage: their proteolytic and lipolytic activity, together with the reduction of nitrates, influence the color, flavor, and stability of the ripened sausages [104,105,106]. CNS can multiply and consume oxygen, resulting in the decline of the redox potential of fermented sausages. The decrease of the redox potential prevents aerobic bacteria and favors the selection of LAB [99].

In Mediterranean sausages, the LAB consist mainly of homofermentative bacilli, while heterofermentative bacilli are less frequent [105]. LAB are characterized by slow growth during the first days of maturation. At the end of the drying stage, they can reach levels of 10^7^–10^8^ CFU/g and exceed one billion cells at the end of ripening [105,106]. The flourishing of this competitive microflora is mainly due to the metabolism of the added sugars, which in turn produces lactic acid, bacteriocins, and inhibitory metabolites, decreasing the pH of the sausages [107,108,109,110,111,112]. Previous studies have shown that the addition of bacteriocins or specific bacteriocin-producing LAB, in association with other preservatives, can enhance the safety and quality of fermented sausages [113,114,115]. 

Throughout the fermentation stage, starters and protective cultures control the ecology of foods through so-called “selfish behavior” [116]. This drive towards a harsh environment for unwanted pathogens and spoilage microorganisms allows the manufacturing of fermented sausages characterized by elevated composition benchmarks [117]. LAB are very significant for the safety of short-ripened sausages, which are not highly dried. In long-ripened sausages, nitrites are reduced, and LAB gradually disappear. Conversely, redox potential and pH rise [99]. Only the a_w_ hurdle is reinforced with time, becoming predominantly responsible for the microbial safety of long-ripened fermented sausages [101]. The combination of sequential hurdles prevents pathogens and spoilage microorganisms in the innermost part of fermented sausages, while the presence of unwanted molds on the outermost layer of the sausages is prevented by smoking or using desirable mold starter cultures [99].

## 4. *Listeria monocytogenes* in Fermented Sausages

Depending on the fermentation conditions, *L. monocytogenes* can persist until the end of ripening or through the storage and marketing of dry-fermented sausages [118]; even so, *L. monocytogenes* tend to decline greatly during the ripening stage [119]. However, despite the “hurdle technology”, the pathogen can become resident in the fermented sausage processing plants and weaken their safety [40,91,100,120]. The contamination of fermented meat products by *L. monocytogenes* may occur at many points of the technological process. The raw meat may be contaminated at the slaughterhouse, during the manufacturing, or by contact with unclean surfaces or workers [52,79] in the post-processing phases [121]. Therefore, the safety of fermented sausages is mainly determined by the management of the fermentation stage [122]. Previous authors have reported occurrence of up to 40%–45% of *L. monocytogenes* in ripened sausages [40,74,85,87,91,123,124,125,126,127,128]. Fermented sausages have hardly ever been associated with notable listeriosis outbreaks [30]. However, some critical outbreaks of food-borne listeriosis related to fermented foods have been reported [129]. Even though there is great diversity among the local traditions that affect the management of fermentation and ripening [12], several Mediterranean-style dry-fermented sausages belong to the group of RTE foods where the survival of *L. monocytogenes* is not assisted. Despite this, in the production of several traditional sausages, empirical management of the “hurdle technology” happens sometimes; for instance, many FBOs are inclined to abbreviate the ripening stage to raise profits for their business [119]. The pH and a_w_ of fermented sausages at the end of ripening are frequently below the levels necessary for the survival of *L. monocytogenes* [12]. However, not enough dried sausages present a_w_ values close to 0.92–0.94 [91] and *L. monocytogenes* may persist during the fermentation stage, overcoming the various hurdles included in the production process [119]. In a recent study [130], weight loss, pH, and a_w_ were estimated as management tools at the ripening stage and used together with predictive mathematical models to assess the persistence of *L. monocytogenes* in fermented sausages. The decrease of a_w_ to safe levels (<0.92) was proved to be closely related to persistence and was confirmed as an easy and useful ripening management tool. *L. monocytogenes* can replicate in fermented sausages with 12 day ripening only when the values of a_w_ are not less than 0.92 [98]. A correct drying process (from 12 to 20 days) is necessary to decrease the a_w_, which can reduce the potential of *L. monocytogenes* survival (<0.92) [98,119,128]. In general, in properly ripened fermented sausages, *L. monocytogenes* cannot compete with the prevailing LAB; therefore, the contamination levels of the pathogen are always ≤100 CFU/g [131]. If antagonistic microflora are lacking, *L. monocytogenes* can grow up to >1000 CFU/g, constituting a primary public health problem [8,52,132]. The most frequent serogroups found in ripened fermented sausages are 1/2c, 1/2a, and 1/2b [52,84,85,91], while serogroup 4b is rare [133,134].

## 5. HHP Processing Technology

HHP processing is a relatively new, non-thermal method of preserving and sterilizing foods, in which a product already sealed in its final flexible and water-resistant package is processed under very high hydrostatic pressure transmitted by water, leading to the elimination of several microorganisms and enzymes from foods [4]. HHP processes subject foods to an elevated degree of isostatic pressure (300–600MPa/43,500–87,000psi) at room temperature for a few seconds to a few minutes [135]. Although the non-thermal pasteurization outcomes of HHP on food products have been known since the 19th century, it was not until the 1990s that the first HHP food products were developed [135]. Since 2000, HHP processing has been successfully implemented in all types of food industry worldwide [135]. At present, technological HHP processing of foods is performed in a batch or semi-continuous treatment; solid food products can only be processed in a batch mode, whilst liquid products can also be processed by means of continuous or semi-continuous treatment [136]. After packaging, food products are laid down in a specifically intended pressure chamber, which is closed and loaded with potable water. A pump joined to the pressure chamber pressurizes the water, (i.e., hydrostatic pressure). The pressure is then transmitted to the food products through the packaging via the water [135]. The isostatic pressures applied to the foods are transferred immediately and are equally distributed; therefore, the treatment is not determined by the form or the extent of the food product [137]. Since the pressure is uniform, the shape is not affected and there is no clear breaking result on the packaging of foods [138]. The pressure is then spread for a fixed time interval, commonly a few seconds but sometimes up to 20 minutes. Once the set interval is completed, the chamber depressurizes and the foods can be taken out [135]. The main benefit of HHP is that the foods are treated regularly all over, which can be difficult to achieve in thermal processing of big or voluminous foods [135].

High pressurization of liquid or solid food products at room temperature is commonly followed by a modest temperature rise (3–6 °C for every 100 MPa increase in pressure), which is called adiabatic heating and is dependent on the food composition [139]. Food products chill down to the initial temperature upon decompression. No heat is misplaced or accumulated throughout the pressure hold interval [136,140].

Using HHP processing to kill bacteria has many advantages: in usual HHP operations, the high hydrostatic pressure spread to food products at room temperature will decrease the levels of most vegetative bacteria by up to 4 log units or greater, and inactivate certain enzymes with just tiny changes in the composition of foods [139,140]. The effectiveness of HHP processing depends on the pressure used, the holding interval, the temperature, the type of food, and the target microorganism [135,140]. Nevertheless, the resistance of bacteria and viruses to HHP is extremely inconsistent, e.g., some Gram-positive microorganisms such as *L. monocytogenes* can present greater resistance than Gram-negative microorganisms such as *Salmonella.* Spores of both bacteria and molds are highly resistant to HHP inactivation. The resistance of viruses is mainly dependent on their morphological structure [139,140,141].

## 6. *Listeria monocytogenes* Inactivation Kinetics in Meat Products via HHP Processing Technology

Frequently used food protection methods are essentially assessed based on their capacity to eliminate foodborne pathogens, thereby enhancing safety and broadening the shelf life of foods through the eradication of spoilage bacteria. HHP has a well-defined benefit in this regard, yielding food products of first-rate quality and nutritional value compared to thermally processed foods [137]. Previous authors have tried to guarantee the HHP inactivation of intentionally spiked or naturally present *L. monocytogenes* in several cured meat products [8,19,20,21,22,24,49,50,142,143].

### 6.1. Poultry Meat Products

Chen et al. [48] showed the outcomes of HHP for the elimination of *L. monocytogenes* in turkey breast meat. Vacuum-packaged turkey samples were treated at several processing combinations (300 MPa/2 min, 400 MPa/1 min, and 500 MPa/1 min at 1, 10, 20, 30, 40, 50, 55 °C). A treatment of 500 MPa/1 min at 40 °C and 20 °C had significant effects on the reduction of *L. monocytogenes* (3.8 log^10^ and 0.9 log^10^ CFU/g, respectively). Patterson, Mackle, and Linton [19] showed the combined outcome of HHP (600 MPa/2 min/20 °C) and sodium lactate (2% w/w) or a pressure-resistant *Weissella viridescens* strain on the elimination of *L. monocytogenes* in spiked (~2.2 × 10^3^ CFU/g) cooked chicken samples stored for up to 105 days at 8 °C. HHP alone was insufficient to inactivate *L. monocytogenes* (>10^8^ CFU/g at day 21), and *W. viridescens* substantially lengthened the lag phase of the *L. monocytogenes* strains that tolerated the HHP processing. In contrast, the association of sodium lactate and HHP successfully inhibited the occurrence of *L. monocytogenes*. Myers et al. [144] studied the survival of *L. monocytogenes* in spiked RTE turkey meat containing sodium nitrite (0 or 200 ppm) and sodium chloride (1.8 or 2.4%), treated at 0 and 600 MPa and stored up to 182 days. HHP (600 MPa/3 min) yielded an inactivation of *L. monocytogenes* between 3.85–4.35 log CFU/g in treated samples. No synergism between HHP and sodium nitrite/sodium chloride on the elimination of *L. monocytogenes* was shown. Stratakos et al. [145] assessed the synergism of coriander oil active film (10%) and HHP (500 MPa/1 min) on the elimination of *L. monocytogenes* in chicken breasts stored for 60 days at 4 °C. The association of coriander oil and HHP showed good synergism in keeping the *L. monocytogenes* count lower than 1.69 log CFU/g. Recently, Balamurugan et al. [24] assessed the influence of salt on HHP elimination of *L. monocytogenes* in pre-blended ground chicken samples. Formulations with NaCl, KCl, and CaCl_2_ at three concentrations (0, 1.5, and 2.5%) and contaminated with *L. monocytogenes* (10^8^ CFU/g) were treated with HHP (0, 100, 300, and 600 MPa for 60 or 180 s). HHP at 100 or 300 MPa and the salts did not significantly decrease *L. monocytogenes* population. Only at 600 MPa was a synergism between salt and duration of HHP processing on *L. monocytogenes* inactivation shown. Formulations with increasing concentrations of CaCl_2_ produced a significantly greater *L. monocytogenes* inactivation rate.

### 6.2. Cooked Pork Hams

The synergism between enterocins A and B, sakacin K, nisin, and potassium lactate against spiked *L. monocytogenes* in sliced cooked ham treated with HHP (400 MPa) was evaluated by Jofré et al. [146]. HHP decreased *L. monocytogenes* (4 log CFU/g) in all samples including bacteriocins. In contrast, HHP decreased *L. monocytogenes* in the control and lactate samples, with counts slowly raising to 6.5 log CFU/g at the end of storage. Marcos et al. [147] evaluated the effectiveness of HHP (400 MPa/10 min/17°C) and enterocin active packaging (200 AU/cm^2^) on the inactivation of *L. monocytogenes* in spiked cooked ham (10^4^ CFU/g) stored for 90 days at 6 °C with temperature abuse. HHP and enterocin successfully precluded the growth of *L. monocytogenes* and kept it at undetectable levels (5 CFU/g) in comparison with untreated samples (8.2–8.8 log CFU/g). In another study, the same authors [148] studied the outcome of HHP (400 MPa/10 min) in association with enterocin and lactate–diacetate on the presence of *L. monocytogenes* in cooked ham stored for 84 days at 1 or 6 °C, and in presence of temperature abuse. Low temperatures, natural antimicrobials, and HHP caused a decrease of 2.7 log CFU/g of *L. monocytogenes*. In another study, the joined outcomes of nisin (800AU/g), potassium lactate (1.8%), and HHP (600 MPa/5 min/10 °C) on the inactivation of *L. monocytogenes* in spiked, vacuum-packed sliced cooked ham (4 log^10^/g) stored for 3 months at 1 and 6 °C was evaluated by the same authors.

The results highlighted that HHP and low temperatures can be successful at challenging survival of *L. monocytogenes* [149]. The shelf-life prolongation of sliced cooked hams treated with HHP (200 and 400 MPa/10 min/17 °C) and enterocin (256/2560 AU/g), stored for 3 months at refrigerated temperature was studied by Liu et al. [150]. The combination of enterocin and HHP (400 MPa/10 min/ 17 °C) was successful in preventing the growth of *L. monocytogenes* and widening the shelf life of treated samples at 90 days in comparison with control samples. More recently, Bover-Cid et al. [151] evaluated the elimination of spiked *L. monocytogenes* (10^7^ CFU/g) in HHP-treated (600 MPa/3 min) sliced standard cooked hams. Standard hams were produced with organic acids and control samples without organic acids, but including potassium lactate and sodium diacetate. The inactivation of *L. monocytogenes* was evaluated in vacuum-packaging storage at 8, 12, and 20 °C. In the samples with potassium lactate and sodium diacetate, the growth of *L. monocytogenes* was barely extended by HHP. In standard cooked hams produced with organic acids, HHP significantly stimulated the growth of *L. monocytogenes*; at 20 °C, the growth of *L. monocytogenes* in samples with lactate was up to 4-fold greater than in the untreated products.

### 6.3. Dry-Cured Pork Hams

Bover-Cid et al. [49] conceived and verified a model of the HHP elimination of *L. monocytogenes* on dry-cured hams (347–852 MPa/2.3 to 15.75 min/7.6 at 24.4 °C). The authors showed that pressure and time were the most important variables influencing the elimination of *L. monocytogenes.* Stollewerk et al. [152] showed the role of salt and efficacy of HHP (600 MPa/5 min) in inactivation of *L. monocytogenes* (spiked at <100 CFU/g) in standard NaCl (28 g/kg) and NaCl-free (replaced with 15.31/kg KCl and 33.83/kg potassium lactate) sliced smoked dry cured hams stored at refrigeration temperature for 112 days. HHP successfully inactivated *L. monocytogenes* in standard samples after 14 days. In contrast, in NaCl-free samples, *L. monocytogenes* was persistent until 28 and 56 days, respectively. In another study, the same authors [153] manufactured three different dry-cured hams (non-acidified smoked, acidified, and acidified smoked) according to a standard and a NaCl-free recipe. Slices of all the dry-cured hams were inoculated with *L. monocytogenes* (<2 log CFU/g), treated with HHP (600 MPa), and stored for 112 days. *L. monocytogenes* was only inactivated in HHP-treated acidified smoked standard ham slices.

NaCl-free hams displayed delayed inactivation of *L. monocytogenes*. In another study, Bover-Cid et al. [22] modeled the inactivation of *L. monocytogenes* in dry-cured ham, in relation to HHP (347–852MPa/5 min/15 °C), a_w_ (0.86–0.96), and fat quantity (10–50 %). The high fat level protected *L. monocytogenes* against HHP at pressure levels of ~700 MPa. At lower pressures, greater elimination of *L. monocytogenes* was shown when the fat level was raised over 30%. In another study, two different RTE dry-cured hams (a_w_ 0.92, 14.25% fat and a_w_ 0.88, 33.26% fat) stored for 60 days at 8 °C were treated with HHP (600 MPa/6 min) with or without use of nisin (200 AU/cm^2^) and examined for the elimination of *L. monocytogenes* (spiked at 10^7^ cells/g). The inactivation of *L. monocytogenes* (from 1.85 to 3.58 log) was high in samples supplemented with nisin. Moreover, a_w_ showed an antagonistic r effect in the elimination of *L. monocytogenes*: with low a_w_ values the inactivation rates were limited [20].

### 6.4. Other Meat Products

Jofré et al. [154] showed the effects of HHP (600 Mpa/6 min/31 °C) in protracting the shelf life of marinated beef loins stored for 120 days. HHP was successful in the elimination of *L. monocytogenes*, which was always below the detection limit in comparison with control samples. HHP elimination (from 300 to 800 MPa) of *L. monocytogenes* (10^7^ CFU/g) on mortadella was developed by Hereu et al. [155]. A clear tail shape was shown at pressure values of >450 MPa. At the uppermost degrees (i.e., 727 and 800 MPa), the *L. monocytogenes* population declined near to the tail level. The high fat level of mortadella (∼17%) had a preserving effect on *L. monocytogenes* from HHP. The same authors [21] evaluated the growth of freeze-stressed or cold-adapted *L. monocytogenes* strains in mortadella inoculated T at 10^7^ and 10^4^ CFU/g) before HHP processing (400 MPa/5 min), and thereupon stored at 4, 8, and 12 °C. Freeze-stressed strains were more resistant to HHP and displayed a greatly extended lag phase following HHP processing. The high fat level composition of mortadella affected the survival of *L. monocytogenes* after HHP treatment. Valdramidis, Patterson, and Linton [23] developed an extended Doehlert model to simulate cured meat treated with HHP (450–800 MPa) and supplemented with sodium chloride/nitrite, as well as the a_w_ values (0.95–0.98). The presence of *L. monocytogenes* was affected by HHP, and HHP showed synergism with a_w_ towards preventing the recovery of *L. monocytogenes*. The concentrations of sodium chloride/nitrite obliquely influenced the occurrence of *L. monocytogenes* by managing the a_w_ values and, therefore, the shelf life of the samples. The lower the a_w_ value, the lower were the inactivation rates caused by HHP.

## 7. *Listeria monocytogenes* Inactivation Kinetics in Fermented Pork Sausages by HHP-Processing Technology

In recent years, the implementation of HHP has been proposed to improve the safety and protract the shelf life of Mediterranean-style dry-fermented sausages (Table 1), since thermal processing may negatively affect their quality. Marcos, Aymerich, and Garriga [156] manufactured typical Spanish low-acid fermented sausages (named “fuet” and “chorizo”) to assess the joined effect of HHP and ripening on inactivation of *L. monocytogenes*. Raw sausages were treated at 300 MPa for 10 min at 17 °C, and subsequently ripened at 12 °C for 27 days.

A decrease in *L. monocytogenes* population in treated “fuet” from 2.26 log CFU/g to 1.42 CFU/g at the end of ripening was reported. In contrast, no effects in *L. monocytogenes* count were noticed in treated “chorizo” samples after 27 days of ripening. The authors highlighted that higher pressure degrees were necessary to eradicate *L. monocytogenes* cells in ripened fermented sausages. Jofré, Aymerich, and Garriga [157] evaluated the use of extra hurdles such as bacteriocins and/or HHP (400 MPa) in low-acid fermented sausages (“fuet”) spiked with *L. monocytogenes* (3 log CFU/g).

Enterocins A and B displayed a prompt decrease of *L. monocytogenes*. In contrast, the application of HHP at the end of ripening did not yield the same result. Despite this, an increasing reduction of *L. monocytogenes* count was noticed throughout storage at room temperature. In another study, Porto-Fett et al. [18] assessed the efficacy of fermentation, drying, and HHP to eliminate spiked *L. monocytogenes* (about 7.0 log CFU/g) in “Genoa” salami. Elimination of *L. monocytogenes* after fermentation ranged from roughly 1.1 to 1.3 log CFU/g. After drying, HHP at 600 MPa or at 483 MPa for 1 to 12 min at 19 °C decreased *L. monocytogenes* enumeration by a further 1.6 to ≥5.0 log CFU/g. After storage for 28 days at 4 °C, *L. monocytogenes* enumeration dropped by up to a further 3.0 log CFU/g. Ananou et al. [17] investigated the single and combined effects of enterocin AS-48 (148 AU g) and HHP (400 MPa for 10 min at 17 °C) on *L. monocytogenes* inactivation in the Spanish sausage “fuet” throughout ripening for 10 days at 15 °C and storage at 7 °C or at room temperature. AS-48 produced a substantial (5.5 log CFU/g) decline in *L. monocytogenes* population. After pressurization and storage, *L. monocytogenes* numbers stayed below 5 CFU/g in all “fuet” samples, including AS-48 (treated or not).

HHP only had no anti-*L. monocytogenes* outcome. Stollewerk et al. [158] evaluated the synergism between the QDS process^®^ and HHP (600 MPa/5 min/13 °C) on inactivation of *L. monocytogenes* in NaCl-free acid (pH 4.8) and low-acid (pH 5.2) “chorizo” stored at refrigeration temperature for 91 days. HHP resulted in the inactivation of *L. monocytogenes* during the whole storage time. The reduction of NaCl could influence the safety of reformulated sausages. However, HHP could be a useful method to develop healthy and safe short-ripened, NaCl-free sausages. Marcos et al. [159] produced sodium-salt-free fermented sausages and spiked *L. monocytogenes* (10^7^ cells/g) onto the outer layer of sausages. The combined outcome of HHP (600 MPa/ 5 min/12 °C) and antimicrobial wrapping with polyvinyl alcohol layers including nisin was evaluated for elimination of *L. monocytogenes* throughout a storage time of 90 days at 4 °C.

The results of this study showed that HHP alone did not preclude the rise of *L. monocytogenes* throughout the storage time. Only the antimicrobial wrapping of fermented sausages with films including nisin caused an evident decrease of *L. monocytogenes* counts (1.4 log CFU/g). Association of HHP and nisin did not yield any extra protection against *L. monocytogenes* compared to nisin wrapping alone. Recently, Rubio et al. [160] implemented a central composite design to assess the outcome of a_w_ (0.79–0.92), pressure (349–600 MPa at 18 °C), and duration (0–12.53 min) on HHP-treated *L. monocytogenes* eradication in Spanish “chorizo” sausages. All the three components greatly affected HHP elimination of *L. monocytogenes*, as the pressure and holding time of HHP treatments increased.

Slight degrees of a_w_ showed a preserving effect on *L. monocytogenes*, and pressures below 400 MPa did not drive towards considerable pathogen decreases. More recently [143], a stochastic simulation modeling approach to evaluate the degree of *L. monocytogenes* occurrence on sliced “chorizo” treated or not with HHP after post-process contamination was developed. The effects of dissimilar degrees of *L. monocytogenes* initial contamination in the minced meat (−1.43–3 log CFU/g), HHP (400–600 MPa/18 °C/0–12 min) and nitrite amounts (0–150 ppm) on *L. monocytogenes* inactivation were evaluated through predictive models, existing references, and experimental data. In all the simulated outlines, compositions, and storage parameters, the population of *L. monocytogenes* on sliced vacuum-packed “chorizo” at the marketing stage was assessed to be below 100 CFU/g, and HHP at 600 MPa for 10–12 min was acceptable in non-contaminated samples.

## 8. Conclusions

The frequent detection of *L. monocytogenes* in Mediterranean-style ripened sausage emphasizes the necessity for the FBOs to implement a multi-hurdle strategy with HHP to enhance the safety of dry-fermented sausages [141]. The HHP elimination of *L. monocytogenes* in fermented sausages is greatly dependent on the physicochemical parameters of the sausages. To define specific pressure treatments accomplishing the essential performance criteria, assessment and validation of the efficacy of HHP on specific Mediterranean-style fermented sausages is imperative [143]. The use of HHP processing technology should help Mediterranean FBOs to comply with European regulations on presence and enumeration of *L monocytogenes* in RTE meat products, as well as optimize the processing conditions of dry-fermented sausages.

## Figures and Tables

**Table 1:** *Listeria monocytogenes* inactivation kinetics in HHP-processed fermented sausages.

Product	HHP Processing Technology	Ripening Time	Inactivation of *L. monocytogenes* by HHP	Multi-Hurdle Approach	Reference
“Fuet” and “chorizo” (Spain)	300 MPa/10 min/17 °C	12 °C/27 days	from 2.26 log CFU/g to 1.42 CFU/g in fuet	None	Marcos et al., 2005 [156]
Fuet (Spain)	400 MPa	Not defined	<1 log CFU/g at the end of ripening	Enterocins A, B and HHP	Jofrè et al., 2009 [157]
“Genoa” salami (Italy)	600 MPa or 483 MPa/1 to 12 min/19 °C	17 °C/25–35 days	1.1 to 1.3 log CFU/g (after fermentation) 1.6 to ≥5.0 log CFU/g (after drying) 3.0 log CFU/g (after storage for 28 d at 4 °C)	Fermentation, drying, and HHP	Porto Fett et al., 2010 [18]
Fuet (Spain)	400 MPa /10 min/17 °C	15 °C/10 days	None	Enterocin AS-48 and HHP	Ananou et al., 2010 [17]
NaCl-free acid (pH 4.8) and low-acid (pH 5.2) chorizo (Spain)	600 MPa/5 min/13 °C	Not defined	<1 log CFU/g at the end of ripening	QDS process® and HHP	Stollewerk et al., 2012 [158]
Sodium-salt-free fermented sausages	600 MPa (5 min/12 °C	Not defined	None	HHP and antimicrobial packaging with films containing nisin	Marcos et al., 2013 [159]
Chorizo (Spain)	349–600 MPa/0–12.53 min/18°C	14–15 °C /10 days	2.47 log CFU/g at 600 MPa	HHP and water activity	Rubio et al., 2018 [160]
Chorizo (Spain)	400–600 MPa/0–12 min/18 °C	14–18 °C/21–42 days	≤1 CFU/g of *L. monocytogenes* in 100% of sausages at the time of consumption (at 600 MPa/10–12 min)	HHP and nitrite	Possas et al., 2019 [143]
